# Enzymatic decontamination of paraoxon-ethyl limits long-term effects in planarians

**DOI:** 10.1038/s41598-020-60846-1

**Published:** 2020-03-02

**Authors:** Laetitia Poirier, Laure Plener, David Daudé, Eric Chabrière

**Affiliations:** 1 Aix Marseille Université, IRD, APHM, MEPHI, IHU-Méditerranée Infection, Marseille, France; 2 Gene&GreenTK, 19-21 Boulevard Jean Moulin, 13005 Marseille, France

**Keywords:** Enzymes, Environmental biotechnology, Environmental impact

## Abstract

Organophosphorus compounds (OP) are highly toxic molecules used as insecticides that inhibit cholinesterase enzymes involved in neuronal transmission. The intensive use of OP for vector control and agriculture has led to environmental pollutions responsible for severe intoxications and putative long-term effects on humans and wild animals. Many *in vivo* models were studied over the years to assess OP acute toxicity, but the long-term effects are poorly documented. Planarian, a freshwater flatworm having a cholinergic system, has emerged as a new original model for addressing both toxicity and developmental perturbations. We used *Schmidtea mediterranea* planarians to evaluate long-term effects of paraoxon-ethyl at two sublethal concentrations over three generations. Toxicity, developmental perturbations and disruption of behavior were rapidly observed and higher sensitivity to paraoxon-ethyl of next generations was noticed suggesting that low insecticide doses can induce transgenerational effects. With the view of limiting OP poisoning, *Sso*Pox, an hyperthermostable enzyme issued from the archaea *Saccharolobus solfataricus*, was used to degrade paraoxon-ethyl prior to planarian exposure. The degradation products, although not lethal to the worms, were found to decrease cholinesterase activities for the last generation of planarians and to induce abnormalities albeit in lower proportion than insecticides.

## Introduction

Insecticides are widely used for vector control to limit disease transmission as well as for pest control in veterinary applications or in agriculture to protect crops^1^. Among these, organophosphorus compounds (OP) are widespread and account for 30% of the global insecticide market^2–4^. These highly toxic molecules target neuronal transmission by inhibiting cholinesterase enzymes including acetylcholinesterase (AChE) and butyrylcholinesterase (BChE). The intensive use of OP in agriculture has led to the pollution of soils and effluents causing many intoxications every year and are suspected to be associated with developmental disturbances and long term effects upon chronic exposure^5–8^. According to the World Health Organization, OP are responsible for millions of intoxications, mainly in developing countries with a mortality rate higher than 15%, and some studies have highlighted an increased sensitivity of pregnant women and children to these compounds^9–13^. Several studies suspect that the presence of OP insecticides in the environment is associated with severe perturbations of wild animals from abnormal behaviors to death^14–17^. To further address the poisoning effects of OP and identify the molecular determinants of their toxicity, various animal models have been considered over the years in the laboratory including mammalian such as guinea pigs or rats and aquatic organisms such as zebrafish^15,18,19^. Although many studies focused on the acute toxicity, the effects of a chronic exposure are still poorly understood due to the lack of appropriate animal model^20,21^. Nevertheless, various effects on skin, eyes, cardiovascular system, gastrointestinal tract, reproductive system and endocrine system could be associated with a chronic exposure to pesticides^9,22^. For example, in rats, a long term exposure to chlorpyrifos, a widespread OP insecticide, induced hypothyroidism of pups^23^. Recently, a new aquatic animal model has emerged for the evaluation of toxicity of exogenous compounds: the planarian, a flatworm belonging to Platyhelminthes phylum. This worm, harboring a mammalian-like cholinergic nervous system, is an excellent animal model to study, not only toxicity, but also developmental disruptions, thanks to a huge proportion of stem cells that confers an extraordinary capacity for regeneration^24–27^. Such an animal model is highly attractive to study both the toxicity of insecticides and evaluate the efficiency of decontamination methods.

Considering the tremendous toxicity of OP, many efforts have been dedicated to the development of decontamination strategies for counteracting their poisoning effects. Enzymatic methods, acting in a smooth and environmentally friendly manner, have emerged to overcome the limitations of chemical methods, including sodium hydroxide or bleaching agents, which are usually pollutant, corrosive and toxic. Although enzymes are very attractive for depollution, their use has been hindered by their low stability that considerably hampered their development. To circumvent this limitation, special interest was drawn to the robust enzyme *Sso*Pox, issued from the archaea *Saccharolobus solfataricus* isolated from the hot springs of Vesuvius. *Sso*Pox belongs to the phosphotriesterase-like-lactonase family (PLL), structurally and biochemically close to bacterial phosphotriesterases (PTE) able to efficiently degrade OP compounds^28,29^. Recently, *Sso*Pox was rationally engineered and led to the isolation of variant *Sso*Pox-αsD6 with proficient capacity to degrade a broad panel of OP^30–34^. The *in vivo* decontamination efficiency of *Sso*Pox-αsD6 was previously demonstrated by protecting planarians from severe exposure to OP^26,35^.

In this study we used planarians to investigate the chronic toxicity of paraoxon-ethyl, the active metabolite of an insecticide parathion-ethyl, at two sublethal concentrations and we evaluate the capacity of *Sso*Pox-αsD6 to decrease poisoning effects in *Schmidtea mediterranea*. Five cholinesterases were first identified and their expression pattern was determined and found altered upon paraoxon-ethyl exposure. Cholinesterase activities, mortality, regeneration process and behavior were then assessed for three generations of planarians and the benefits of enzymatic decontamination was emphasized.

## Results and Discussion

### Identifying and localizing expression of *S. mediterranea* cholinesterases

**Figure 1:**
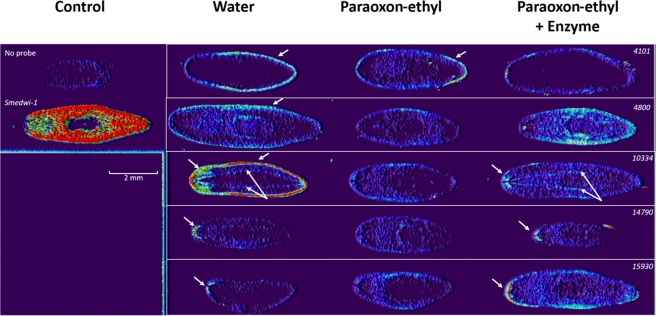
Localization of planarian cholinesterases by WISH (Whole-mount *in situ* hybridization) processed by ImageJ. Smedwi-1 is a marker of proliferation cells. All cholinesterase numbers were retrieved from PlanMine database and specified in the right corner in italics. Head of planarians are always located on the left. Distinct nervous system patterns were identified with white arrows. The impacts of paraoxon-ethyl (5 µM, 1/2 of NOEC) and enzyme decontamination on cholinesterase expression were determined. Scale bar represents 2 mm.

Planarians were previously shown to harbor cholinesterases sharing common ancestors with human enzymes^26,36^. Nevertheless, the number of planarian cholinesterases and their respective roles are poorly understood. Here, we report for the first time the presence of five putative distinct cholinesterases in *S*. mediterranea (*4101, 4800, 10334, 14790* and 15930) that were predicted using PlanMine database. To localize the expression pattern of these cholinesterases, whole *in situ* hybridization (WISH) was performed **(**Fig. 1 and Supplementary Fig. 1). Interestingly, all cholinesterase expression patterns differ from each other suggesting different putative functions for planarian cholinesterases. Cholinesterases are mainly expressed in the anterior part in the cephalic ganglion (*10334, 14790* and 15930), all along the body following ventral nerve cords (10334) and at the external border of the planarian (*4101, 10334*, and 15930). The strongest gene expressions were obtained for 10334 which expression localizes along the planarian nervous system^37,38^ and for 4800 with a more diffused expression pattern all along the body. In another planarian specie, *Dugesia japonica*, two cholinesterase genes, namely *djche1* and *djche2* were previously reported^36^: *djche1* has the same expression profile as *smed_10334* and protein Djche1 share 74.84% identity at the protein level whereas Djche2 shares 68.55% identity with Smed_4800 and both genes are ubiquitously expressed throughout the worm body.

Impact of paraoxon-ethyl exposure at sublethal concentration (5 µM, 1/2 of NOEC) on cholinesterase expressions was assessed. WISH results highlighted that paraoxon-ethyl exposure decreased cholinesterase expression level except for 4101 for which the cholinesterase expression of the external border of the planarian was preserved (Fig. 1).

To evaluate the benefits of enzymatic decontamination of paraoxon-ethyl, planarian cholinesterase expressions were evaluated after treatment by *Sso*Pox-αsD6. This variant was previously described to efficiently degrade a broad spectrum of organophosphorus insecticides including paraoxon-ethyl with a catalytic efficiency of 2.95 ± 1.55 × 10^4^ M^−1^.s^−1^ allowing a fast and complete decontamination and the corresponding degradation products were characterized^26,34^. The enzyme appeared to protect planarian cholinesterase expression in most cases **(**Fig. 1): the expression of 4101, 4800 and 14790 was comparable to control condition in water, expression of cholinesterase 10334 was less affected after enzymatic treatment than for paraoxon-ethyl, and the cholinesterase 15930 exhibited a different pattern upon enzymatic treatment than in water with a stronger expression at the external border as compared to control. Paraoxon-ethyl is known to inhibit cholinesterase activities, the inhibition of cholinesterase expression may be an indirect effect due to acetylcholine accumulation or non-cholinergic effect of paraoxon-ethyl, nevertheless this effect can be prevented by enzymatic degradation.

### Long-term exposure of planarian to sublethal concentrations of paraoxon-ethyl

**Figure 2:**
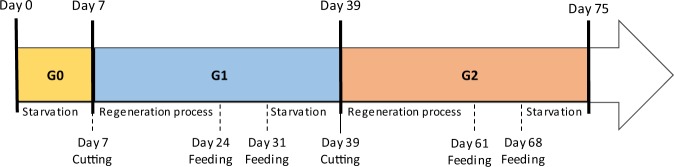
Schematic timeline of long-term exposure of planarians. Starvation time and regeneration process are 7 days and 14 days respectively. The experiment was conducted over 75 days. Several checkpoints were analyzed in the course of the experiment (survival, mobility and regeneration process) and cholinesterase activities were evaluated at the end of each generation.

To evaluate the consequences of chronic exposure in planarians at sublethal doses of paraoxon-ethyl, a long-term experiment was conducted by exposing three generations of worms to insecticide concentrations of 5 µM and 0.5 µM corresponding to 1/2 and 1/20 of the NOEC determined for intact worms (Supplementary Fig. 2). To further evaluate the benefits of enzymatic decontamination, experiments were also performed after degradation of paraoxon-ethyl by *Sso*Pox-αsD6 (Supplementary Fig. 3). Survival rates, planarian locomotion, behavioral effects and cholinesterase activities were thus analyzed for the three generations, with and without enzymatic treatment (Fig. 2).

### Impact of paraoxon-ethyl on cholinesterase activities and protective effect of decontaminating enzyme

**Figure 3:**
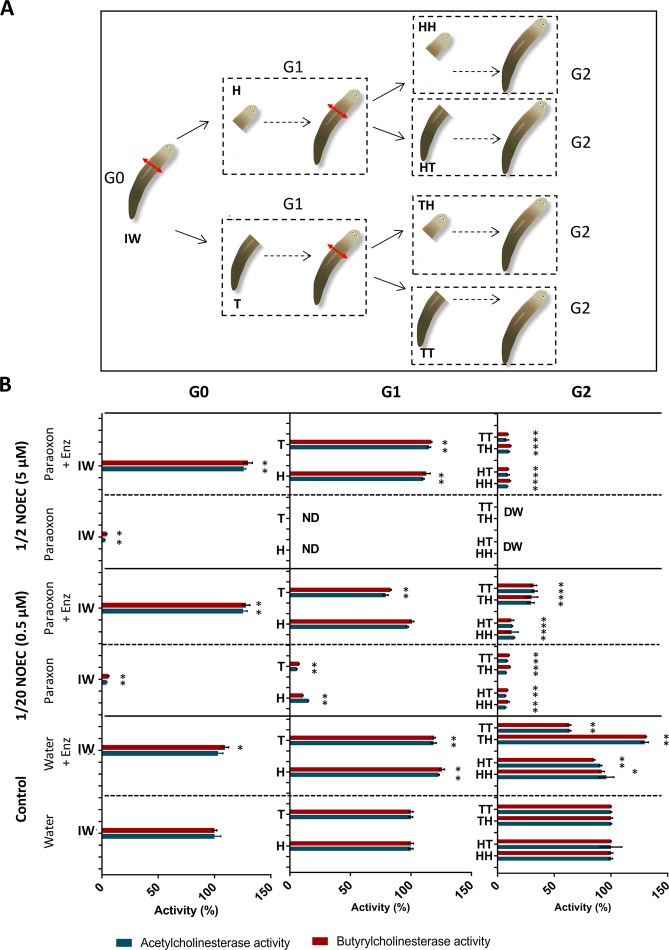
Evaluation of planarian cholinesterase activities over generations. (**A**) Schematic representation of planarian generations (intact worm (IW), head (H), tail (T)) (**B**) Blue and red bars represent acetylcholinesterase and butyrylcholinesterase activity respectively (no determined data (ND), dead worm (DW)). The values represent the mean ± SD (standard deviation) of three replicates. Black stars (*) indicate a significant difference between the condition studied and the corresponding control in water according to Tukey’s test.

To determine the impact of paraoxon-ethyl on cholinergic activity of planarians, acetylcholinesterase and butyrylcholinesterase activities were measured for each generation using acetylthiocholine and butyrylthiocholine as substrates **(**Fig. 3A). For G0 and G1 generations, cholinesterase activities were inhibited for both concentrations used for paraoxon-ethyl exposure (1/2 and 1/20 NOEC corresponding to 5 µM and 0.5 µM respectively), whereas cholinergic activities were protected after enzymatic degradation (Fig. 3B). At the highest paraoxon-ethyl concentration (1/2 NOEC), planarians were not viable and cholinesterase activities were not measurable at G1, while upon enzymatic decontamination worms still survived at G2 although their cholinesterase activities were decreased as compared to the control in water. At the lowest paraoxon-ethyl concentration (1/20 NOEC), cholinesterase activities were inhibited at G2 with or without enzymatic treatment, albeit to a lesser extent upon enzymatic decontamination. Conversely to G1, enzyme treatment did not fully protect cholinesterase activities at G2 suggesting a long-term sensitization to paraoxon-ethyl over regenerating worms. These results were consistent with previous *in vitro* observations reporting AChE activity reduction down to 40% after paraoxon-ethyl exposure at sublethal concentration^26^. Interestingly, although cholinesterase expression was mainly detected in the anterior moiety of the worms, no difference was observed between cholinesterase activities from anterior and posterior fragment after paraoxon-ethyl exposure.

### Evolution of survival of planarians upon paraoxon-ethyl exposure

**Figure 4:**
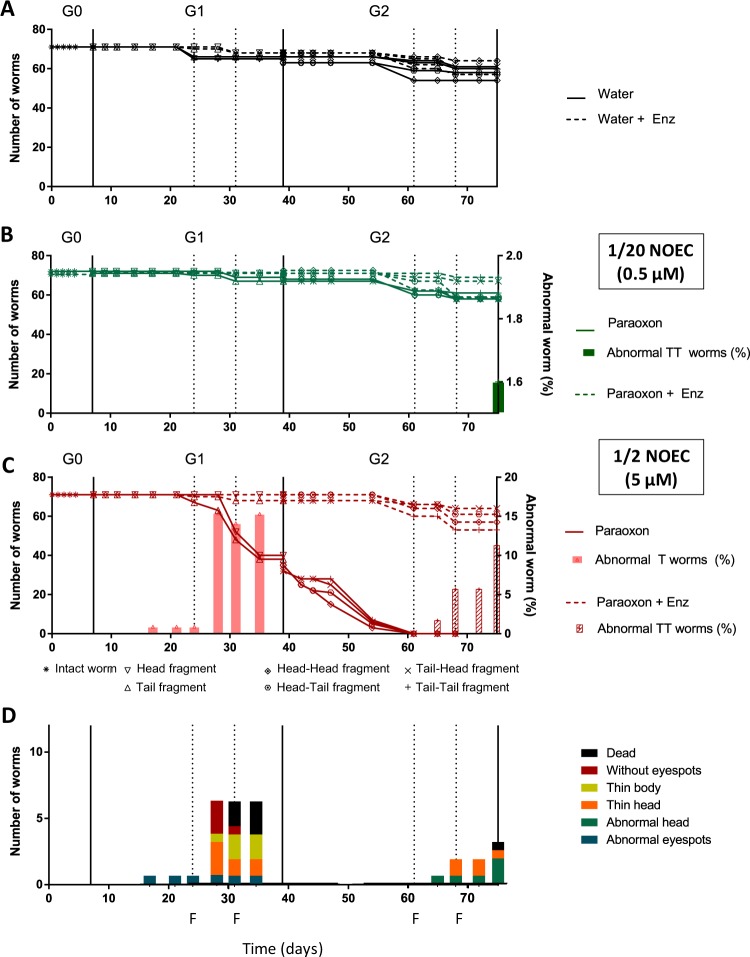
Survival rates in planarians over generations. The dotted lines correspond to the presence of the enzyme *Sso*Pox-αsD6. (**A**) Control conditions in water and enzyme solution. (**B**) Paraoxon-ethyl at 0.5 µM (1/20 NOEC) (full lines) and corresponding degradation products (dotted lines). One abnormal worm was observed with abnormal tail at day 75 for paraoxon-ethyl tail-tail fragment. (**C**) Paraoxon-ethyl at 5 µM (1/2 NOEC) (full lines) and corresponding degradation product (dotted lines). Abnormal worms are indicated with full histograms for paraoxon-ethyl and dotted histograms for the degradation products. (**D**) Planarian abnormal phenotypes are described over the experiment. Feeding steps are indicated by the letter F. T stands for tail.

Paraoxon-ethyl concentrations, 1/2 and 1/20 of the NOEC value, were chosen because they do not induce any observable effects on planarians during a first exposition. The toxicity of paraoxon-ethyl during the two next generations of planarians was assessed by measuring the survival rates of worms with or without enzymatic decontamination (Fig. 4).

For G0, as expected, after 7 days of exposure, no mortality was observed for both sublethal concentrations studied.

Exposure to the highest concentration of paraoxon-ethyl (1/2 NOEC) was fatal to approximatively 50% of worms at the end of G1 (day 39), the other half of the worms being close to death (Fig. 4C). Besides mortality, up to 15.9% of planarians developed morphological abnormalities. All abnormal planarians were not viable for the next generation. After enzymatic degradation of paraoxon-ethyl at 1/2 NOEC, no toxicity was observed (Fig. 4C). When exposing planarians to the lowest concentration of paraoxon-ethyl (1/20 NOEC), no impact on planarian survival was observed with or without enzymatic treatment (Fig. 4B).

For G2, exposure to 1/2 NOEC of paraoxon-ethyl led to complete mortality of worms after 61 days (Fig. 4C), while after enzymatic degradation, planarians were still alive although morphological abnormalities could be observed for 11.3% of planarians from day 65 until the end of the experiment (Fig. 4C). When exposing planarians to 1/20 NOEC of paraoxon-ethyl, no toxicity was noticed except in one planarian showing abnormal tail at the end of the generation without enzymatic treatment (day 75) (Fig. 4B).

Altogether, these results confirmed that sublethal concentrations of paraoxon-ethyl induce chronic effect in planarians, particularly for the highest concentration (1/2 NOEC) and that enzymatic decontamination showed remarkable protective effects on mortality although some abnormalities could be observed at the end of G2 for a small proportion of worms (Fig. 4B).

Although cholinesterase expressions were decreased after paraoxon-ethyl exposure (Fig. 1) planarians were still alive underlining more complex mechanisms involved during OP exposure. Another study highlighted that long term exposure to chlorpyrifos, diisopropylphosphorofluoridate and parathion of rats decreased the level of synaptic variant AChE mRNA^39^. Altogether, these results suggest that AChE gene expression could be associated with long-term behavioral effects of OP.

### Paraoxon-ethyl induces developmental perturbations of planarians while enzyme decontamination shows protective effect

**Figure 5:**
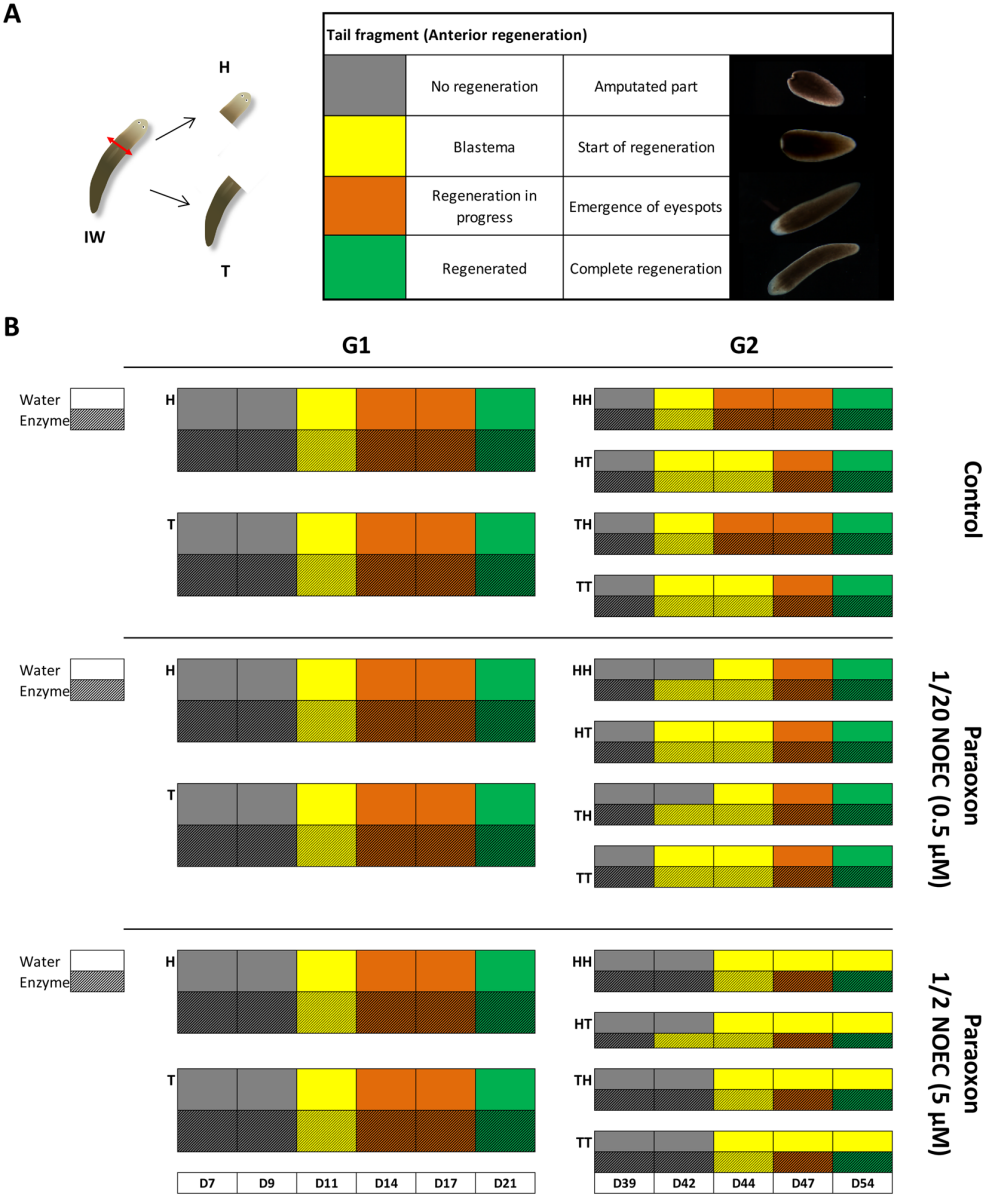
Planarian anterior regeneration process over generations. The striped colors correspond to the presence of the enzyme *Sso*Pox-αsD6. (**A**) Planarians were cut above the pharynx using a scalpel (intact worm (IW), head (H), tail (T)). Head regeneration of planarians is presented using a color code from grey to green describing initial to final regeneration stages. Posterior regeneration is described in Supplementary Fig. 4. (**B**) Regeneration process monitoring for each condition: control conditions in water and enzyme solution, paraoxon-ethyl at 0.5 µM and its degradation product and paraoxon-ethyl at 5 µM and its degradation product.

Planarians, with a large amount of stem cells, have an unconventional capacity to regenerate an entire worm from a tissue fragment in 15 days^26^. To evaluate whether sublethal concentrations of paraoxon-ethyl may induce developmental disruption in planarians, regeneration was monitored over long-term exposure. A categorization based on physical characteristics was used for determining regeneration stages from no development to a complete regeneration (Fig. 5A and Supplementary Fig. 4). Normal regeneration process was observed for G1 in all conditions (Fig. 5B). Conversely, G2 regeneration was drastically altered upon exposure to the highest concentration of paraoxon-ethyl (1/2 NOEC) and the regeneration stopped after the emergence of the blastema for each fragment (HH, HT, TH and TT) until planarian death. Upon enzymatic degradation of paraoxon-ethyl (1/2 NOEC) the regeneration was complete with only a slight delay as compared to the control. A similar regeneration profile was observed at 1/20 NOEC of paraoxon-ethyl, with and without enzymatic treatment, with a 2 days delay of regeneration as compared to control conditions.

**Figure 6:**
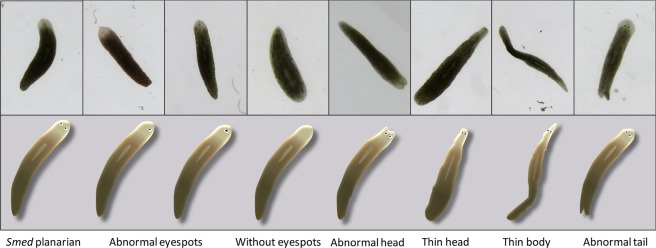
Pictures and illustrations of planarian abnormal phenotypes observed during experiment.

As previously noticed (Figs. 4D and 6), abnormal worms were observed for G1 and G2. Surprisingly, abnormalities only occurred after complete regeneration of the worms and several phenotypes were noticed from thin head to missing eyespots (Figs. 4D and 6 and Supplementary Fig. 5). Interestingly, all abnormal worms were issued from tail fragments suggesting that anterior regeneration process is more sensitive to paraoxon-ethyl than posterior regeneration, correlating with cholinesterase expression patterns, although no difference in the cholinesterase activity of the two fragments was noticed (Fig. 3**)**. These observations suggest that anterior and posterior regeneration processes are differently affected by paraoxon-ethyl exposure. The mechanisms generating these differences still remain to be understood and would benefit from thorough comparisons of expression and activity profiles of cholinesterases over the course of regeneration. Planarian anterior alterations were previously reported by silencing genes, especially *Smedwi-2*, known to be involved in stem cells proliferation, or resistance to irradiation^40,41^. However, the abnormalities of eyespots reported in these studies appeared earlier in the regeneration process than in our study. Nevertheless, the capacity of paraoxon-ethyl to interfere with the stem-cell based regeneration process in planarian would require further investigation. In addition, exposures to exogenic compounds like ivermectin, a drug used to treat many types of parasite infestations, and glyphosate, the active ingredient of the widespread herbicide Roundup, have led to similar abnormalities in planarians in recent studies^42,43^.

### Evaluation of behavioral disruptions in planarians

**Figure 7:**
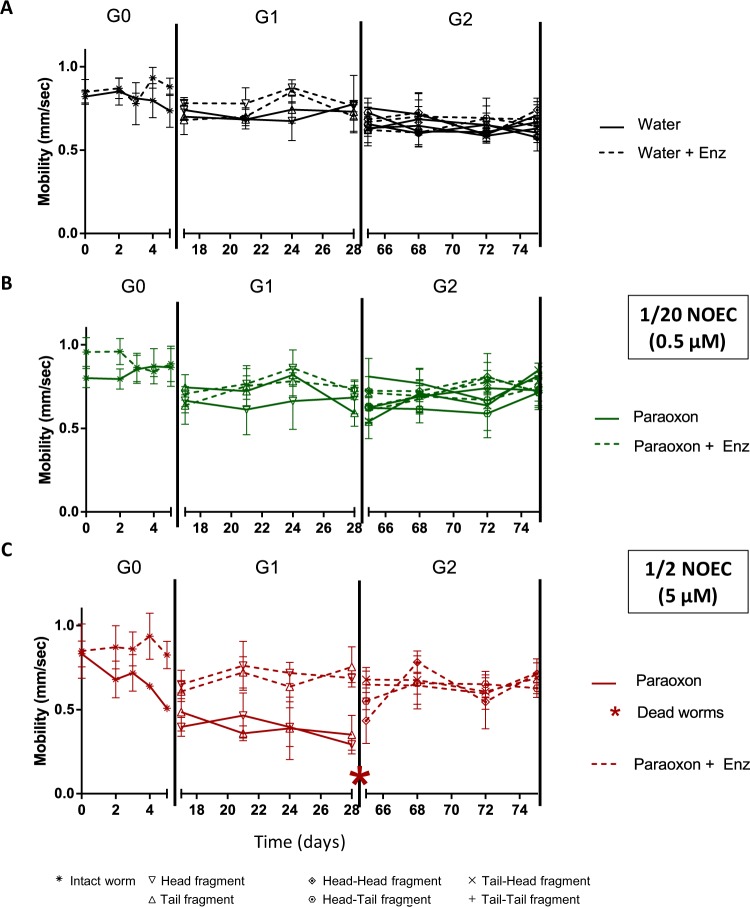
Planarian mobility over generations. The dotted lines correspond to the presence of the enzyme *Sso*Pox-αsD6. The values represent the mean ± SD (standard deviation) of three distinct planarians. (**A**) Control conditions in water and enzyme solution. (**B**) Paraoxon-ethyl at 0.5 µM and corresponding degradation product. (**C**) Paraoxon-ethyl at 5 µM and corresponding degradation product. The red stars indicate the dead worms.

OP intoxications were previously shown to induce behavioral disruption in planarians^24,26,44^. Here, we evaluated long-term behavioral disruptive effects for low paraoxon-ethyl concentrations over generations. Normal planarian mobility was approximatively 0.8 mm/sec (Fig. 7A). For exposure at 1/2 NOEC of paraoxon-ethyl, planarian mobility started to decrease at the end of G0 and was reduced down to approximatively 0.3 mm/sec during G1 (Fig. 7C). After enzymatic degradation, no disruption of behavior was observed under any conditions. For G2 generation, the mobility of planarians exposed to paraoxon-ethyl at 1/2 NOEC was dramatically altered prior to the death of the worms (Fig. 4C), whereas planarians were as mobile as controls for the other conditions. Paraoxon-ethyl exposure at 1/20 NOEC, with and without enzymatic treatment, did not impair planarian mobility neither for G1 nor G2 generations (Fig. 7B).

Paraoxon-ethyl was previously showed to induce acute toxicity in several animal models including planarians^15,26,35^. Here we proved that sublethal concentrations of paraoxon-ethyl, lower than the NOEC values determined during acute exposure, induced chronic effects in next generations of planarians. For the highest insecticide concentration (5 µM), abnormalities and mortality were observed from G1 generation and complete mortality was observed at G2. These results clearly demonstrate that exposure to low concentration with no mortality at G0 was lethal for the next generation. When using enzymatic decontamination, complete degradation of paraoxon-ethyl was achieved. The long-term effects in planarians was drastically reduced as compared to insecticide exposure. Nevertheless, we found that the degradation products, although they were not lethal to the worms, induced abnormalities at G2 generation for 5 µM and led to cholinesterase inhibition at both concentrations suggesting toxic effects of paraoxon-ethyl degradation products on planarians (Figs. 3B and 4C). These results were unexpected as the degradation products of paraoxon-ethyl are widely believed not to inhibit cholinergic receptors. Special attention should thus be paid to the evolution of shortly-persisting organophosphorus compounds in the environment as their degradation products could induce long-term effects in living organisms.

## Materials and Methods

### Production and purification of *Sso*Pox-αsD6

pET32b-Δtrx plasmid containing the *Sso*Pox-αsD6 coding sequence was transformed in *E. coli* BL21(DE3)-pGro7/GroEL (TaKaRa) chaperone expressing strain as previously described^26,30,34^. Briefly, the production was performed in an auto-inducible ZYP medium (complemented with 100 µg.ml^−1^ ampicillin and 34 µg.ml^−1^ chloramphenicol) at 37 °C. Addition of CoCl_2_ and L-arabinose (final concentrations of 0.2 mM and 2 g.l^−1^ respectively) was used to induce the expression of the chaperones GroEL/ES with a decrease in temperature overnight at 23 °C. Then, mechanic and enzymatic lysis were performed and enzyme *Ss*oPox-αsD6 was purified in two steps of size exclusion chromatography (HiPrep 26/10 desalting, GE Healthcare; ÄKTA Avant and HiLoad 16/600 Superdex 75 pg, GE Healthcare; ÄKTA Avant). The purity of the enzyme was checked by 12.5% SDS-page and final concentration was determined using a NanoDrop 2000 spectrophotometer (Thermo Scientific).

### Planarians

Asexual *Schmidtea mediterranea* freshwater worms (asexual clonal line ClW4) were used in all experiments. The planarians were stored in sterile water, without antibiotics, at 19 °C in the dark, fed once a week with calf liver purchased from local butcher. The water was cleaned every two days and healthy planarians were manually selected with a size of approximately 0.8 cm in length. A starvation step was performed 10 days prior to the experiment. Observations were performed using a Leica M165 FC lens connected to the IC Capture 2.4 software and planarian mobility was measured by tracking, using ImageJ software.

### Planarian cholinesterase identification

To identify planarian homologs of *Homo sapiens* cholinesterases, a TBLASTN analysis of the *Schmidtea mediterranea* planarian transcriptome database PlanMine (http://planmine.mpi-cbg.de/planmine/begin.do) was performed using acetylcholinesterase isoform E4-E5 precursor (NP_056646.1) and butyrylcholinesterase precursor (NP_000046.1) sequences^45^. Seven sequences producing a significant alignment with the genes of interest were identified. No additional cholinesterase on PlanMine was identified. Multiple sequence alignment of planarian cholinesterases were performed using Clustal 2.1^46^ and five potential distinct planarian cholinesterases were identified: dd_Smed_v6_4101_0_1, dd_Smed_v6_4800_0_1, dd_Smed_v6_10334_0_1, dd_Smed_v6_14790_0_1 and dd_Smed_v6_15930_0_1, the two other sequences were redundant with previous ones (dd_Smed_v6_4800_0_1 and dd_Smed_v6_15930_0_1) (Supplementary Table 1). The presence of the catalytic triad was verified (*Hs*_AChE: SEH) and found conserved in 4 out 5 sequences, except dd_Smed_v6_15930_0_1 (NEQ) (Supplementary Fig. 6). Structural predictions were performed with Phyre² (http://www.sbg.bio.ic.ac.uk/phyre2/html/page.cgi?id=index) and were found closely related to the template c4bdA_ corresponding to human acetylcholinesterase in complex with huprine w and fasciculin 2 (Supplementary Fig. 6). Confidence of 100% and coverage between 76% for dd_Smed_v6_15930_0_1 and 96% for dd_Smed_v6_4101_0_1 were obtained.

### Whole-mount *in situ* hybridization (WISH)

WISH was performed as previously described^47^. Briefly, worms were fixed, dehydrated and stored in methanol 100% at −20 °C prior to their use. A pre-treatment was performed including a bleaching step before labelling. Riboprobes were synthetized with polymerase T7 (Promega) for pPT-T4P-based template using digoxigenin (DIG)-labelling mix (Roche) and were then stored at −20 °C.

Images were collected using a Leica M165 FC lens connected to the IC Capture 2.4 software (Supplementary Fig. 1) and were processed using ImageJ software by subtracting background (50 pixels, light background and sliding paraboloid) (Fig. 1). Contrasts were enhanced by a 0.1%-fold and interactive 3D surface plot was performed with the following parameters: grid size 1024, smoothing 0, perspective 0.2, lighting 0.38 and z-scale 0.10 (min: 37%, max: 82%).

### Toxicity assays

#### NOEC determination

4 mL of paraoxon-ethyl (Sigma-Aldrich) in water were prepared at different concentrations (10 µM–50 µM). Experiments were performed using 12-well microplates. Five worms were incubated per well and experiments were performed in duplicates. Mortality was followed for 14 days.

#### Chronic exposure

Experiments were conducted using 6-well microplates, with a maximum of 15 worms per well containing 4 mL of solution, at two sub-lethal concentrations of paraoxon-ethyl based on NOEC determination: 1/2 NOEC and 1/20 NOEC. Enzymatic degradation of paraoxon-ethyl was performed by *Sso*Pox-αsD6 (at a final concentration of 0.25 µM, according to previous results^26^) for 3 hours to reach a complete degradation (Supplementary Fig. 3). Planarian generations (G0, G1 and G2, Fig. 2) were studied over a 75-day period. Two cutting steps were performed on day 7 and 39, and feeding steps were performed on days 24, 31, 61 and 68. Each solution was changed after cutting and feeding steps and new 6-well microplates were used. A portion of worms were lost during feeding and cutting steps all along the experiment. Control experiments were performed in planarian water with and without enzyme.

For each generation, planarian mortality and mobility were analyzed. Mobility values represent the mean ± SD (standard deviation) of the mobility of three distinct worms. Abnormal worms were observed for each condition and abnormal worm evolution was monitored over 39 days to observe successive phenotypes on a same planarian (Supplementary Fig. 5).

The regeneration process was followed subsequently to worm amputation with a scalpel just above the pharynx. The head and tail fragments were respectively regrouped in a well and the regeneration of each part was followed using criteria from no regeneration to a complete regeneration (Fig. 5 and Supplementary Fig. 4).

### Planarian cholinesterase activity assays

Acetylcholinesterase and butyrylcholinesterase activities were measured as previously described with acetylthiocholine and butyrylthiocholine as substrates^48^. For each generation, 20 worms were ground in a Fastprep (three cycles of 10 seconds, 6.0 m.s^−1^) in 500 μL, 400 µL and 300 µL of activity buffer (4-(2-hydroxyethyl)−1-piperazineethanesulfonic acid 50 mM, NaCl 150 mM, pH 8) for G0, G1 and G2 respectively. Experiments were performed in the same buffer complemented with 4 mM 5,5′-Dithiobis (2-nitrobenzoic acid) in a 96-well microplate and OD_412nm_ was recorded using a microplate reader (Synergy HT,BioTek, USA) for 10 minutes. Negative controls were performed without planarian homogenate. Activities were normalized with the water control of each condition. For G1 planarian in paraoxon-ethyl at 5 µM, no data was collected because the worms were not viable.

### Statistical analyses

For cholinesterase activities, the values represent the mean ± SD (standard deviation) of the percentage of three technical replicates. Two-way ANOVA was performed with a fixed type I error of 5% by GraphPad Prism 6. The conditions were compared to the control activity in water using the Tukey’s test. According to Tukey’s t-test, the significance was indicated with black stars.

## Supplementary information

Supplementary Information.
