# Supplementary material for: Enzymatic decontamination of paraoxon-ethyl limits long-term effects in planarians
Source: Sci Rep. 2020 Mar 2;10:3843. doi: 10.1038/s41598-020-60846-1 (PMC7052158; doi:10.1038/s41598-020-60846-1)
Supplement: Supplementary file 1 — Supplementary Information. [file 41598_2020_60846_MOESM1_ESM.pdf]

SUPPORTING INFORMATION

Enzymatic decontamination of paraoxon-ethyl limits long-term effects in planarians

Laetitia Poirier[1], Laure Plener[2], David Daudé[2,°], Eric Chabrière[1,°]

[1] Aix Marseille Université, IRD, APHM, MEPHI, IHU-Méditerranée Infection, Marseille, France,

[2] Gene&GreenTK, 19-21 Boulevard Jean Moulin, 13005 Marseille, France.

° Corresponding authors

Supplementary table 1. Multiple sequence alignment of planarian cholinesterases (Smed_4101, Smed_4800, Smed_10334, Smed_14790 and Smed_15930) with acetylcholinesterase isoform E4-E5 precursor (NP_056646.1) and butyrylcholinesterase precursor (NP_000046.1) using Clustal 2.1. Catalytic triad is indicated in red.

Identity (%)	NP_000046.1	NP_056646.1	Smed_4101	Smed_4800	Smed_10334	Smed_14790	Smed_15930
NP_000046.1	-	46.0133	27.3743	25.7475	22.4252	23.588	23.588
NP_056646.1		-	23.2775	23.825	23.1767	23.6629	24.9595
Smed_4101			-	25.5121	22.5326	24.0223	21.9739
Smed_4800				-	29.5238	37.8086	29.7297
Smed_10334					-	24.6032	26.6667
Smed_14790						-	28.3951
Smed_15930							-

CLUSTAL 2.1 multiple sequence alignment

```
Smed_4800        MKRFRRQMVFKGSLLIIKLIFIYRIIYINSISGETFKKSILIKEKRYELLGLTLNIY-GK
Smed_14790       ----MRVEFEASSLIVLCFCLSYDAIVVH----------IKIDNQTFPLKGKSIFKM-GK
NP_000046.1      --------MHSKVTIICIRFLFWFLLLCMLIGKSHTEDDIIIATKNGKVRGMNLTVF-GG
NP_056646.1      -MRPPQCLLHTPSLASPLLLLLLWLLGGGVGAEGREDAELLVTVRGGRLRGIRLKTP-GG
Smed_15930       -------------MEFWSYSLWIFVWVTRGLTFNRNGEYVTVSTTNGNIMGIKKDVL-GK
Smed_10334       ----------MEFVSFIVLLTAYSAVSYPITPNYTPSVRTKIGTINGFISKVEWNDRSAT
Smed_4101        ---------------------------------------MLNFSVSNGLVQGQTRKVLHR
                                                                 :

Smed_4800        TIDHFARIPYALPPINNLRFKYPQEIVGNQWKGVYNATTSPNTCWQVE-GGEFDKLNPAA
Smed_14790       KIFHFSKIPYAKPPIGNRRFRYPEKLNNPPWTGVYDSTVQPLTCWQGAPSIEFELKNPIG
NP_000046.1      TVTAFLGIPYAQPPLGRLRFKKPQSL--TKWSDIWNATKYANSCCQNIDQSFPGFHG--S
NP_056646.1      PVSAFLGIPFAEPPMGPRRFLPPEPK--QPWSGVVDATTFQSVCYQYVDTLYPGFEG--T
Smed_15930       PVYVFLGIPYAKPPVGRLRFRNPELVN--KWEGTYSATKLPKTCYQNLPEHYELNNP-AW
Smed_10334       TVHIYYGIPYAIPPVKNLRFKKPVPFP-EISEKVVNAQTHKPSCHQYNDTSYLNTAG--A
Smed_4101        DIAEFLSIPYCKPPIGELRFQKPQPFS--SFSKSIINRGKFDTCPPQLPMRIFNGENLDE
                  :  :  **:. **:   **  *                    *

Smed_4800        RLWLSKTPMSEDCLYLTIWSPRT-----------VKTKLPVMVWIYGGSFFSGSSTLEVY
Smed_14790       KMWFSKTVMSEDCLYLTIWTPQINV---------ANELLPVMIWIYGGSYMSGSSTLEVY
NP_000046.1      EMWNPNTDLSEDCLYLNVWIPAPK----------PKN-ATVLIWIYGGGFQTGTSSLHVY
NP_056646.1      EMWNPNRELSEDCLYLNVWTPYPR----------PTSPTPVLVWIYGGGFYSGASSLDVY
Smed_15930       RIWTNNTEMSEDCLYLSIWTPKINKNQNRKM-FNEKFIISTLVWIHGGGFTGGSSTLDIY
Smed_10334       RMWVSPIPFDEDCLYLNIWVPQNPAPDVHKMNQKTTDKLAVMVWIYGGSFASGAAGLEVY
Smed_4101        ETWIPKERQDENSLYVNVWCPLEILK--------EKENQPVLVWIYGGGYVSGSSSLEIY
                 . *      .*:.**:.:* *              .   ..::**:**.:  *:: *.:*

Smed_4800        DGSILAAKHEVVVVSLQYRLGPLGFLYLDDTL-APGNQGLMDQRMALKWVKDNIYNFNGD
Smed_14790       DGSILASLHNVVIVSFQFRSGPLGFLYLDDTE-SPGNQAIMDQHMLLKWVNNHIVSFGGD
NP_000046.1      DGKFLARVERVIVVSMNYRVGALGFLALPGNPEAPGNMGLFDQQLALQWVQKNIAAFGGN
NP_056646.1      DGRFLVQAERTVLVSMNYRVGAFGFLALPGSREAPGNVGLLDQRLALQWVQENVAAFGGD
Smed_15930       NGEILASKMNVIVVNIQYRLGPFGFLSY-NVKEIPGNQGLMDQVLALQWIRDNIGFFGGD
Smed_10334       EGRYLAARQNVIVVSMNYRLGPFGFIYLKNGL-IPGNMGLWDQRLALKWVKENIEFFGGD
Smed_4101        DGSVLASKENIIVVSFNYRIAMLGFISLDDEI-LNGNLGIWDQVCALQWIHENISHFKGN
                 :*  *.   . ::*.:::* . :**:   .     ** .: **   *:*:..::  * *:

Smed_4800        SESITIFGESAGSVGVSIHVLSPLSNNLFKRAIMESATASAAWAIETKKESKEKGLAISK
Smed_14790       PKKVTLFGESSGATSISVHLFSKISEKYFQRAILQSGTIFVPWAIESSSASYIKAKYLAE
NP_000046.1      PKSVTLFGESAGAASVSLHLLSPGSHSLFTRAILQSGSFNAPWAVTSLYEARNRTLNLAK
NP_056646.1      PTSVTLFGESAGAASVGMHLLSPPSRGLFHRAVLQSGAPNGPWATVGMGEARRRATQLAH
Smed_15930       SKLVTIFGANSGAVSASFHLLSPLTQDLFNQAIMQSGSALSWWAYDDPKTAISKAYHFAN
Smed_10334       SDKITIFGESAGSVSVSAHVISPWSQNLFRNAIMQSGSVLGYWGLISRNTSEERTKILMN
Smed_4101        PKNVTIVGNSAGGCSVSLHLLIEKSWEYFQNAIMLSGVCLSDWGFLTKNEVRDRSKKAVN
                 .  :*:.* .:*. . . *::   :   * .*:: *.     *.         :     .

Smed_4800        FVNCNYDS----WQVNVLLKCLQKVKPDLLVSKQLDLKNIVNKQRSDLLRRRGVKNLFTD
Smed_14790       KANCTSRN----NQE--IVKCLRSTKPEVLVNLNFLIRDQIAKTRKEKLMKEGYKDFYSD
NP_000046.1      LTGCSRE------NETEIIKCLRNKDPQEILLNEAFVVPYGT------------------
```

```
NP_056646.1      LVGCPPGGT--GGNDTELVACLRTRPAQVLVNHEWHVLPQES------------------
Smed_15930       LSGCHNSKETVEIKTSDLFECLRSLDPITLVVNQWKMLNERYR-------EKLTKPILPY
Smed_10334       RLKCRGSLH-------AKVECLRQLPAKQLTDAHSYLYDANS------------------
Smed_4101        KILEKFDFPMKWNEEITRENKIAIIKFLQNINFKEFLLDTELVTG---------------
                                      :           .  :

Smed_4800        ASFFFDIFFRPTYDANFINQPVNLLLNDSTFKPN-IELLMGYNANEGMFFLLYGLNKWLN
Smed_14790       AALFFDIYFRPIFDQKIISKLDVEKISEGQLVTN-KDLLIGTNANEAMFFLMYGLSSNFK
NP_000046.1      ---PLSVNFGPTVDGDFLTDMPDILLELGQFKK--TQILVGVNKDEGTAFLVYGAPGFSK
NP_056646.1      ---VFRFSFVPVVDGDFLSDTPEALINAGDFHG--LQVLVGVVKDEGSYFLVYGAPGFSK
Smed_15930       ASFYFDIPFKPTIGGLYLPKPPSSYFAGKEPKILRKRVMLGTTKNEGLIHLFKGFRHYFM
Smed_10334       ---YFSVPFPPVIDNHFLPYPNSQAFKNLVHVKPTGALMLGMNKNEGSYFMLYSFIPNSD
Smed_4101        ---ILQFAWAPCVDGELIKDDPRRLFNEKKFKICPTIFLN--VANEGTYFIISRFPNIDV
                     : . : *  .   :       :           .:     :*.  .:.

Smed_4800        FFNKGEVEDTSLLSSNNTNYIKASKFLLENLTPNDIAYTNLLPLLLHEYKIPSTIFNWKK
Smed_14790       LFESSKIVLPSKMSYPSENYTDPYKIVLENILDLDYLYPSLTKLILHEYGIQSQHFDDKS
NP_000046.1      DNNS-------------------IITRKEFQEGLKIFFPGVSEFGKESILFHYTDWVDD-
NP_056646.1      DNES-------------------LISRAEFLAGVRVGVPQVSDLAAEAVVLHYTDWLHP-
Smed_15930       PSDDSLVQPALFRYG--PNTKDPADLLTHYIVNDKFVNHFNIEAIAFEYELPSKIFQQSN
Smed_10334       WLNNKTSVQIAN-----------NKDYQEKLRKVLDLEAKIPSQILSLVDFEYTDYNLP-
Smed_4101        DNHK---------------------MIDNKYFTQTFQYYPHFPIICSQSTRKKISVEYVL
                   ..                        .

Smed_4800        WNSTDVLSALDQLTGDQNFVCPVIDYAELLAD------------KNVKVYLYSFQHRTSR
Smed_14790       WTSKEILQKLDFIGGDMQFICPTIQFADSVTKS-----------KSSKLFMYLFNCRTNG
NP_000046.1      QRPENYREALGDVVGDYNFICPALEFTKKFSE------------WGNNAFFYYFEHRSSK
NP_056646.1      EDPARLREALSDVVGDHNVVCPVAQLAGRLAA------------QGARVYAYVFEHRAST
Smed_15930       WKSQDIINVFDNIAGDFSFKCPVVDFADKYLK-------------IAQVYLYSFEQQTQF
Smed_10334       DTPQNRVNRLEEISSDRSFKCPTIEMAKMVNNDNRFEKGLGKRSVTLPTYFYEFRYRTKS
Smed_4101        NEPSLSIDILDRIIADQYFISGSIELANLISKS------------NSKVYYLWFETFTQP
                   .    . :  : .*  . .   : :                      :   *.  :.

Smed_4800        MTFPKWTGTMHGYEIEYVFGMPFSKNFQNLFYTFTTEEAKLSNYIMTLWANFAKFGNPTT
Smed_14790       TEVPDWLGVMHGYEIEYVFGMPFSSDFTSNLYSFNDDEKNVSRSVMKFWTNFAKYGNPTP
NP_000046.1      LPWPEWMGVMHGYEIEFVFGLPLERRDN-----YTKAEEILSRSIVKRWANFAKYGNPN-
NP_056646.1      LSWPLWMGVPHGYEIEFIFGIPLDPSRN-----YTAEEKIFAQRLMRYWANFARTGDPN-
Smed_15930       LPLPPWSGVLQGQEIAYVFGKPFDMEFRKKYYNFSEEEMKLSEKIMSYWTNFAQTGQPNN
Smed_10334       LPWPEWMGTIHGLEIDYVFGVPFNKKFEKIFYEFTDEERRISDVVMQYWANFARHGDPNI
Smed_4101        EHWPNWCGVMHSEEIYYLFGHYLTQQSE-------TRQSATSIKIMKEFGNFVKHGNLSE
                    * * *. :. ** ::**  :              :   :  ::  : **.: *: .

Smed_4800        ----------QKIEYFDSTLSWPLYNTTSKLSVYFTTPHESQLNAARLNDKAMKCKFWNK
Smed_14790       ----------TDFTEKG---FWPAYTNKNKRYLKISG-TNSGGFKVENHFKQKHCRFWTK
NP_000046.1      ------------ETQN-NSTSWPVFKSTEQKYLTLNT----ESTRIMTKLRAQQCRFWTS
NP_056646.1      ------------EPRDPKAPQWPPYTAGAQQYVSLDL----RPLEVRRGLRAQACAFWNR
Smed_15930       R--------MREYHTRTRGMHWDTYSGTNINGRKHLIFRNNRVLAGRN-FRRHECLFWRN
Smed_10334       MPNDKLVSDYDVSINGKPLRVWPEFDNTTQSYIIIKE---DGNLTTSVNPKKRKCLFWRR
Smed_4101        VFR----------------------NYDESSNAYIKINEDLWTSAIFSKDQINKVRFWEK
                                                                  :     **

Smed_4800        VIPPILEKFKNFSPTLNKVAWTNIVIIIIINNTRLTTEEHNKICKNL-------------
Smed_14790       ILPDLLNRLLNKK---NGSSALLLVDSLLVSSVLKVLASFMALCFRSI------------
NP_000046.1      FFPKVLEMTG--------NIDEAEWEWKAGFHRWNNYMMDWKNQFNDYTSKKESCVGL--
NP_056646.1      FLPKLLSATASEAPSTCPGFTHGEAAPRPGLPLPLLLLHQLLLLFLSHLRRL--------
Smed_15930       YLPDMRKNILSNSKCNPRTNTVQKPIVKNPTYTSIQNSSPLIDRPIAKSSKRNPNDDKNN
Smed_10334       WYPILLKETKPKHINCSHRNRNAGLKR---------------------------------
Smed_4101        LRRKFYDNF---------------------------------------------------
                     . .

Smed_4800        --------------------------
Smed_14790       --------------------------
NP_000046.1      --------------------------
NP_056646.1      --------------------------
Smed_15930       KSTIIGIENLLFISIIWIFQMLKYLF
Smed_10334       --------------------------
Smed_4101        --------------------------
```

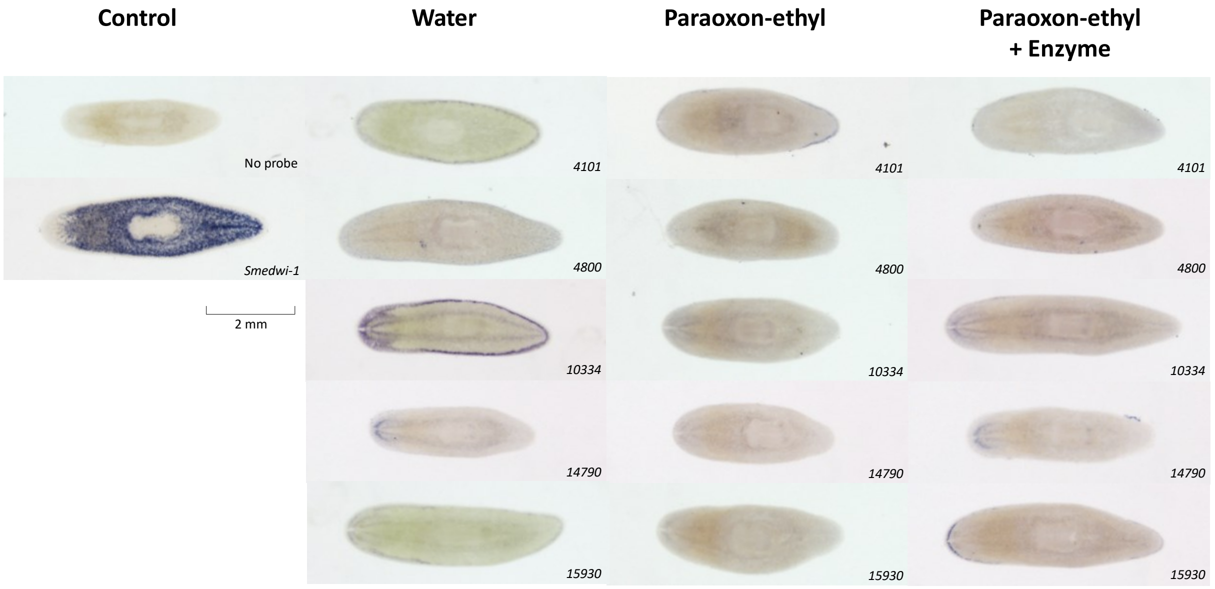

Supplementary figure 1. Localization of planarian cholinesterases by WISH (Whole-mount in situ hybridization). *Smedwi-1* is a marker of proliferation cells. All cholinesterase numbers were retrieved from PlanMine database and specified in the lower right corner in italics for each picture. Distinct nervous pattern systems were identified and impact of paraxon-ethyl (5 µM, 1/2 of NOEC) was determined. Scale bar represents 2 mm.

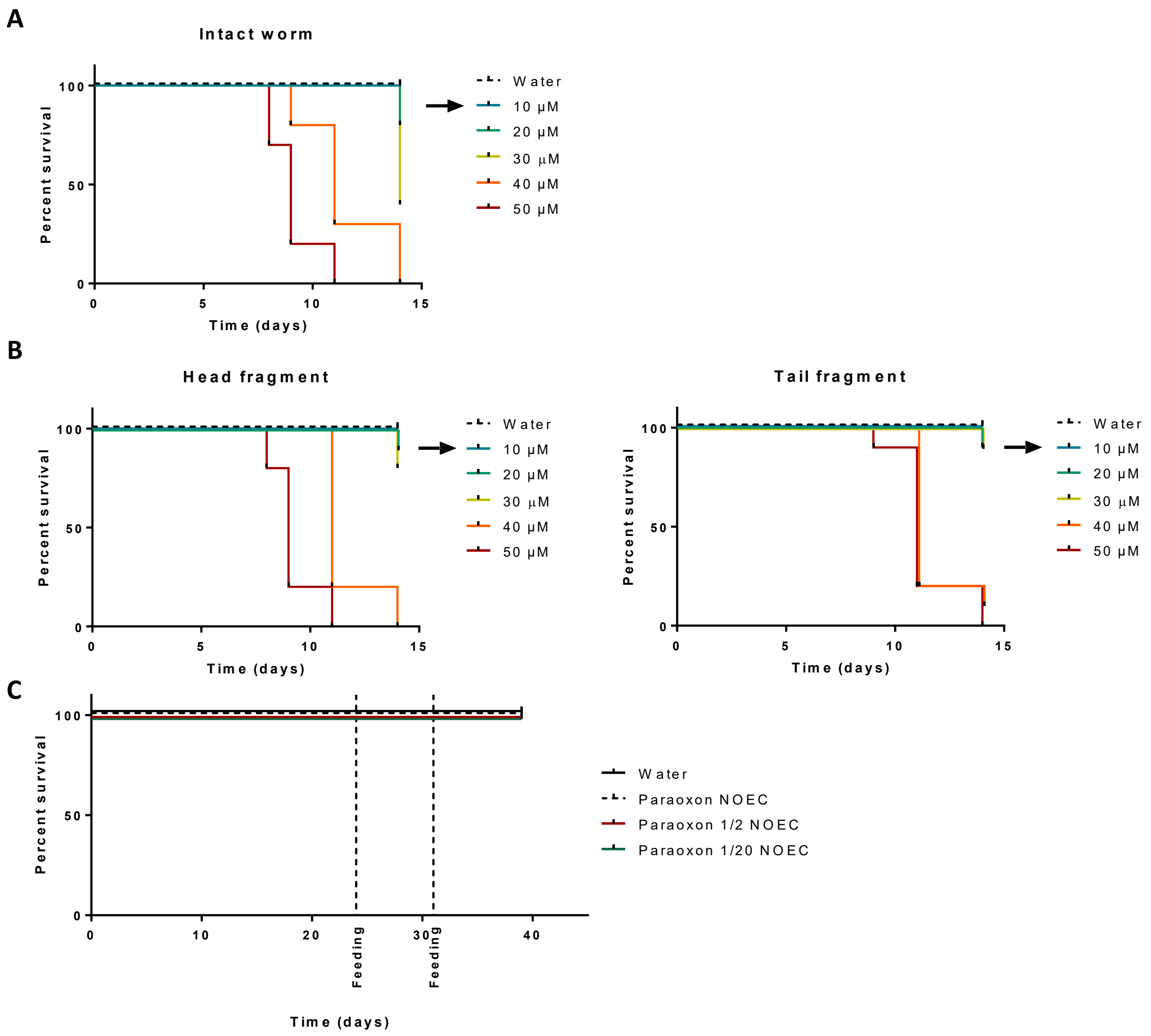

Supplementary figure 2. Survival rates of planarians. The toxicity of paraoxon-ethyl was evaluated for concentrations ranging from 10 µM up to 50 µM for intact planarians (A) and after amputation on head and tail fragments (B). The curves represent the survival rates for 10 planarians exposed to paraoxon-ethyl. Arrows indicate NOEC values. (C) As control NOEC value of 10 µM was verified over 39 days on intact worms.

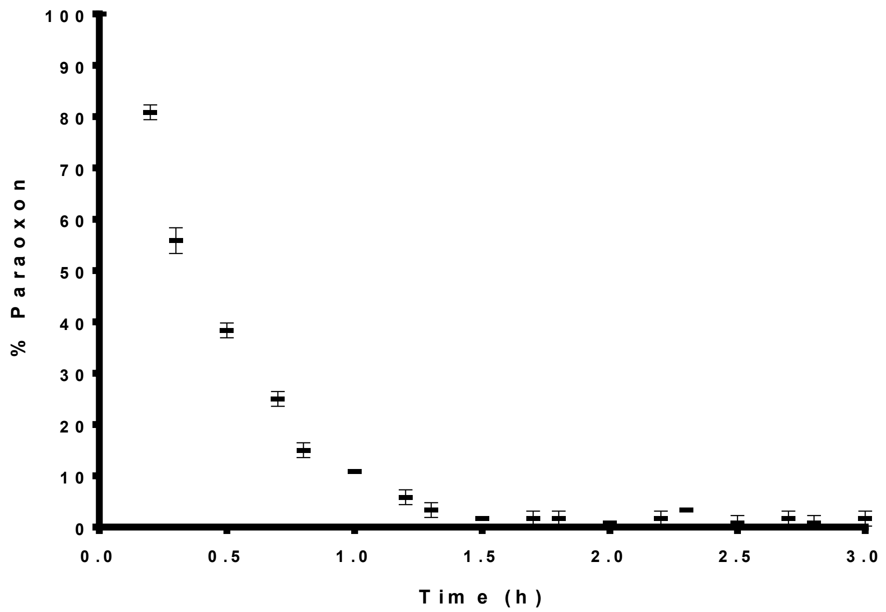

Supplementary figure 3. 5µM Paraoxon-ethyl degradation curve. The degradation of 5µM paraoxon-ethyl by the enzyme *Sso*Pox αsD6 at 0.25µM was followed over time. Full degradation was reached after 1.5h incubation.

Head fragment (Posterior regeneration)			
(grey)	No regeneration	Amputated part	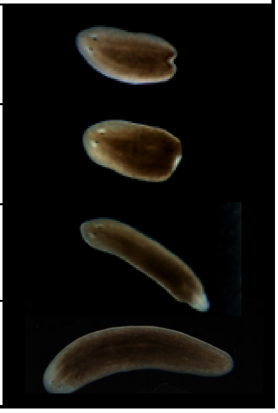
(yellow)	Blastema	Start of regeneration	
(orange)	Regeneration in progress	Emergence of the pharynx	
(green)	Regenerated	Complete regeneration	

Supplementary figure 4. Planarian posterior regeneration is presented using a color code from grey to green describing initial to final regeneration stages.

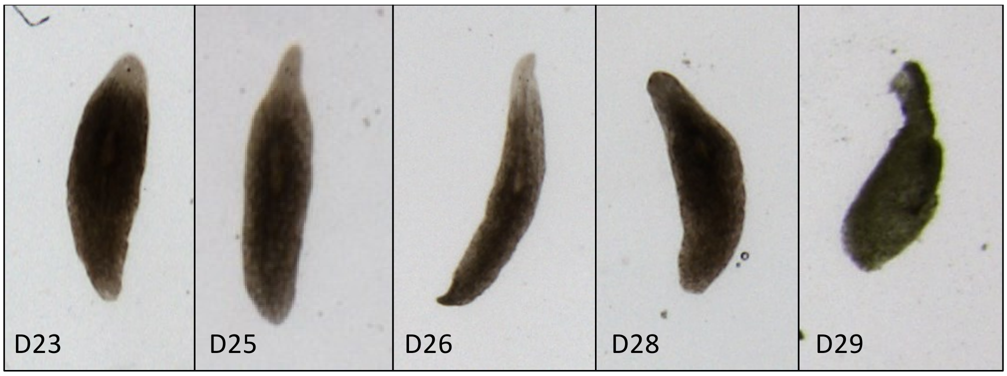

Abnormal eyespots	Thin head	Thin body	Without eyespots	Dead worm

Supplementary figure 5. Temporal evolution of an abnormal worm exposed to paraoxon-ethyl 5 µM. The worm arose from a G1 tail fragment.

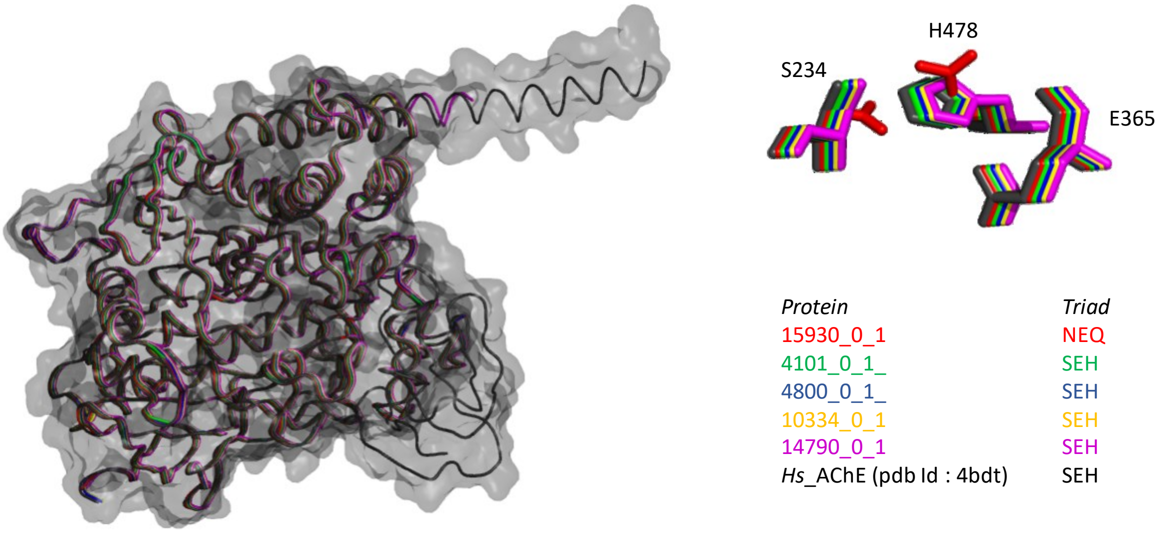

Supplementary figure 6. Structural predictions of planarian cholinesterases compared to the best template c4bdA_ corresponding to human acetylcholinesterase in complex with huprine w and fasciculin 2 (pdb : 4bdt). Conservation of the catalytic triad in all sequences, except dd_Smed_v6_15930_0_1.
